# Synthesis, Quantification, and Characterization of Fatty Acid Amides from In Vitro and In Vivo Sources

**DOI:** 10.3390/molecules26092543

**Published:** 2021-04-27

**Authors:** Ruidong Ni, Suzeeta Bhandari, Perry R. Mitchell, Gabriela Suarez, Neel B. Patel, Kara Lamb, Kirpal S. Bisht, David J. Merkler

**Affiliations:** Department of Chemistry, University of South Florida, 4202 E. Fowler Ave., Tampa, FL 33620, USA; ruidongni@usf.edu (R.N.); suzeeta@usf.edu (S.B.); Perry.r.mitchell@gmail.com (P.R.M.J.); gabriela23@usf.edu (G.S.); neelpatel4@usf.edu (N.B.P.); karalamb@usf.edu (K.L.)

**Keywords:** fatty acid amide, liquid chromatography/quadrupole time-of-flight mass spectrometry, anandamide, N_18_TG_2_, choroid plexus, *Drosophila*, *Bombyx*, *Apis*, *Tribolium*

## Abstract

Fatty acid amides are a diverse family of underappreciated, biologically occurring lipids. Herein, the methods for the chemical synthesis and subsequent characterization of specific members of the fatty acid amide family are described. The synthetically prepared fatty acid amides and those obtained commercially are used as standards for the characterization and quantification of the fatty acid amides produced by biological systems, a fatty acid amidome. The fatty acid amidomes from mouse N_18_TG_2_ cells, sheep choroid plexus cells, *Drosophila melanogaster*, *Bombyx mori*, *Apis mellifera,* and *Tribolium castaneum* are presented.

## 1. Introduction

Fatty acid amides are a family of intriguing, yet structurally simple lipids, R-CO-NH-R’. The acyl moiety, R-CO-, is derived from the fatty acids listed in most undergraduate biochemistry textbooks and the -NH-R’ moiety is derived from the set of biogenic amines. The structural simplicity of the fatty acid amides belies both the importance and diversity of this lipid family. Hundreds of different fatty acid amides are possible and, to date, approximately 90 different fatty acid amides have been identified from living organisms [1]. In vivo, accumulating evidence suggests that the fatty acid amides are cell signaling lipids [2,3,4,5,6] and have a technological use as slip additives in plastics [7].

The biological occurrence of the fatty acid amide bond traces back to the 1880s with the first characterization of sphingomyelin by Thudichum [8]. Decades after the work of Thudichum, *N*-palmitoylethanolamine was isolated from egg yolk [9] and five different primary fatty acid amides were identified in luteal phase plasma [10]. Interest in the fatty acid amides increased dramatically after the identification of *N*-arachidonoylethanolamine (anandamide) as the endogenous ligand of the CB_1_ receptor found in the mammalian brain [11]. Other key discoveries cementing the biological importance of the fatty acid amides were the demonstration that *N*-(17-hydroxylinolenoyl)-l-glutamine (volicitin) as an elicitor of plant volatiles produced in insects [12] and the characterization of oleamide as a regulator of the sleep/wake cycle found in the mammalian brain [13]. Endocannabinoids are endogenous lipid-based ligands that bind to the CB_1_ and CB_2_ receptors [4,5]. The fatty acid amides are, thus, endocannbinoid-like or endocannbinoid related by virtue of their structural similarity to anandamide.

Our interest in the fatty acid amides stemmed from the discovery that peptidylglycine α-amidating monooxygenase (PAM) catalyzes the oxidation of *N*-fatty acylglycines to primary fatty acid amides (PFAMs) [14,15,16]. Our initial focus on the *N*-fatty acylglycines and the PFAMs lead to broader interest in the fatty acid amide family and the identification of *N*-acyltransferases responsible for their biosynthesis [17,18,19]. One aspect of our broader interest in the fatty acid amide family were studies to isolate and quantify the fatty acid amides produced in model organisms, called the fatty acid amidome. We synthesized specific fatty acid amides as standards for the liquid chromatography/quadrupole time-of-flight mass spectrometry (LC/QTOF-MS) method to define the fatty acid amidome [18,20]. In this review, we first describe the synthesis and characterization of specific fatty acid amides and then summarize our results on the characterization of the fatty acid amidome from cultured mammalian cells [15] and insects [18,19,20,21]. The novelty of this article relative to other reviews on the endocannbinoids [1,2,3,4,5,6] is a broad focus on the endocannbinoid-related fatty acid amides, coverage of the fatty acid amides identified in insects, and the methodologies for chemical synthesis of the standards that were used in the characterization of the fatty acid amidome.

## 2. Results and Discussion

### 2.1. Synthesis of the Fatty Acid Amides

**Figure 1:**
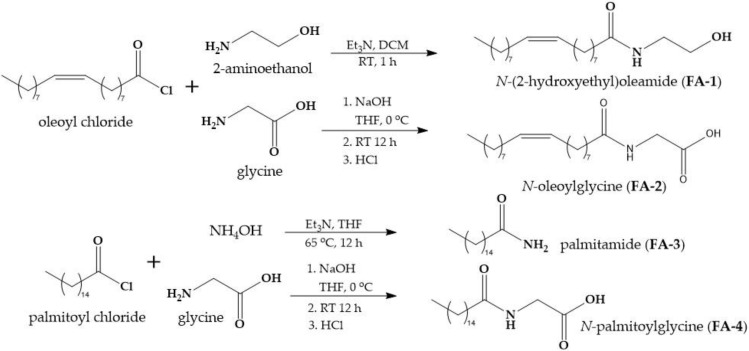
The synthetic strategy for the fatty acid amides.

The synthetic routes and structures of the fatty acid amides prepared in this study are shown in Figure 1. The condensation of the corresponding fatty acid chloride with 2-aminoethanol or glycine in the presence of triethylamine or NaOH, respectively, gave the desired fatty acid amide (**FA-1**, **2**, and **4**) in good to excellent yield [22,23]. Palmitamide (**FA-3**) was obtained by condensation of the palmitoyl chloride with ammonia in the presence of triethylamine [24].

### 2.2. Structural Analysis of the Synthetically Prepared Fatty Acid Amides

The molecular structures of the fatty acid amides **FA-1**, **2**, **3** and **4** were confirmed spectroscopically (^1^H and ^13^C-NMR, and MS; Supplementary Materials Figures S1–S8) and upon comparison to reported literature values. All the obtained fatty acid amides **1**–**4** were white solids. In the ^1^H- and ^13^C-NMR spectra, alkenic (=C*H*) hydrogens in **FA-1** and **FA 2** appeared as a multiplet at 5.3 ppm and the alkenic (=*C*) carbons were observed at 129 and 130 ppm, respectively. The NHC*H_2_* hydrogens in **FA-1** were observed as a triplet of a doublet at 3.4 ppm (J = 4.8 and 5.2 Hz), showing coupling of the NH and the hydrogens of the hydroxymethylene group. The NHC*H_2_* hydrogens in **FA-2** and **FA-4** appeared as a doublet (*J* = 6 Hz) at 3.7 ppm, due to its coupling with the NH hydrogen. The **FA-2** and **FA-4**, in the ^13^C-NMR, showed two resonances corresponding to the amide and carboxylic acid carbonyls at 170 and 171 ppm, for **FA-1** and **FA-3**, the single amide carbonyl resonance was observed at ~174 ppm. Each of the fatty acid amide was also subjected to the high-resolution mass spectrometry, and the exact mass of the compounds was determined to be within acceptable instrumental error.

### 2.3. The Fatty Acid Amidome from Mouse N_18_TG_2_ Cells and Sheep Choroid Plexus (SCP) Cells

Our initial interest in defining the fatty acid amidome was narrowly focused on the PAM-mediated conversion of *N*-fatty acylglycines to the PFAMs. To this end, our first model system was mouse neuroblastoma N_18_TG_2_ cells, cells known to produce oleamide [25] and other fatty acid amides [26]. We demonstrated that these cells express PAM [27], would convert exogenously added oleic acid to oleamide], and that inhibition of PAM activity either by the growth of cells in the presence of a PAM inhibitor [28] or by siRNA resulted in a decrease in oleamide production and an accumulation of *N*-oleoylglycine [16]. These results provided strong evidence for a cellular role of PAM in the PFAM biosynthesis.

Choroid plexus cells are responsible for the production of cerebrospinal fluid (CSF) [29] and express PAM [30]. Since PAM [31] and oleamide [13] are found in the CSF, we chose sheep choroid plexus (SCP) cells as another model system for our work on the fatty acid amidome. The fatty acid amides identified and characterized from the mouse N_18_TG_2_ cells and the SCP cells are shown in Table 1. *N*-Palmitoylethanolamine and *N*-stearoylethanolamine were identified in the neuroblastoma C1300 N18 cells [32], cells related to the N_18_TG_2_ cells. We have refrained from including the published quantification values for the individual fatty acid amides, instead we have employed a +, ++, or +++ system that reflects the relative abundance of each fatty acid amide. The published abundance values often show considerable scatter, examples being the reports for the levels of oleamide from the N_18_TG_2_ values being 530 ± 300 pmoles/10^7^ cells from Jeffries et al. [16] and 55 ± 10 pmoles/10^7^ cells from Bisogno et al. [25]. As discussed by Marchioni et al. [33], the scatter results from the challenges in measuring the relatively low levels of the fatty acid amides from biological samples. Thus, it seems best to conclude that the fatty acid amides are produced by the N_18_TG_2_ cells and the SCP cells and that specific fatty acid amides are produced at higher levels than others. The appropriate references are included in Table 1 for those desiring to see the reported abundance values. The SCP studies were driven by a proposed metabolic connection between the fatty acids, the *N*-acylethanolamines, the PFAMs and only levels of the PFAMs in the SCP were measured. In this focused study, there were no attempts to isolate and quantify fatty acid amides other than the PFAMs [15]. Fatty acid amides have been identified from mammalian sources other than the N_18_TG_2_ and the SCP cells, as described in these reports [3,4,5,34,35].

The cellular function(s) for most of the fatty acid amides are unclear [5,35]; thus, the significance of the differences between the levels of the individual fatty acid amides within a fatty acid amidome and between the fatty acid amidomes of the N_18_TG_2_ and SCP cells are uncertain.

### 2.4. The Fatty Acid Amidome from Insects: Drosophila Melanogaster, Bombyx Mori, Apis Mellifera, and Tribolium Castaneum

We decided to pursue insect model systems for our fatty acid amide studies for a number of reasons because insects are inexpensive to maintain, have defined life cycles, their genomes have been sequenced, and are easy to manipulate genetically. In addition, a cell signaling fatty acid amide, *N*-(17-hydroxylinolenoyl)-l-glutamine (volicitin) [12], had been described from the army beetworm. Insects often express a set of *N*-acyltransferases enzymes that could have a role in fatty acid amide biosynthesis [36]. The lack of cannabinoid receptors in *Drosophila* [37] hinted at an intriguing evolutionary history for the endocannabinoid system if fruit flies produced fatty acid amides.

We found that a number of fatty acid amides were produced by *Drosophila melanogaster* (Table 2 and Supplementary Materials Table S1) [18,38]. Our report came after a publication from Tortoriello et al. [39], showing that 45 different fatty acid amides are produced by *D. melanogaster* larvae (Table 2). With the exception of *N*-linoleoylglycine and *N*-linoleoylethanolamine, Tortoriello et al. [39], did not quantify the fatty acid amides they identified in the *D. melanogaster* larvae, reporting the other fatty acid amides as either detected or not detected. Again, we have refrained from reporting the quantification values for the individual fatty acid amides, but instead use “detected,” “not detected,” or a plus sign (+) to indicate relative abundance. To provide perspective, the fatty acid amide of the highest abundance was *N*-oleoylglycine at 500 ± 300 pmoles/(g of head) [18] while *N*-linoleoylethanolamine was 0.34 ± 0.02 pmole/(g of larvae) [39].

One objective of our fatty acid amide studies in insects was to identify the enzymes involved in their biosynthesis, which might be a target for a novel insecticide. One key to this objective were control insects of benefit to mankind because an effective new insecticide would specifically target insect pests. Two economically important insects are *Apis mellifera* (the honey bee) and *Bombyx mori* (the domestic silkworm). Fatty acid amides were identified and characterized from both insects, the fatty acid amidome for *A. mellifera* is shown in Table 3 and that for *B. mori* is in Table 4. Differences were found between the fatty acid amidomes of the head, thorax, and abdomen in *A. mellifera*, one clear difference being the relatively high amounts of *N*-oleoylethanolamine in the abdomen. The *B. mori* life cycle includes pupae, moth, a pre-instar ant stage, and five instars [40]. The fatty acid amidome of the 4th instar of *B. mori* is less populated relative to the other insect fatty acid amidomes that we have characterized, compare Table 4 to Table 2 and Table 3. The significance of these data is unclear and, ultimately, we plan to characterize the fatty acid amidome for all the lifecycle stages of *B. mori*. Such an analysis could contribute to defining the function of specific fatty acid amides in *B. mori* and other organisms, as well.

**Figure 2:**
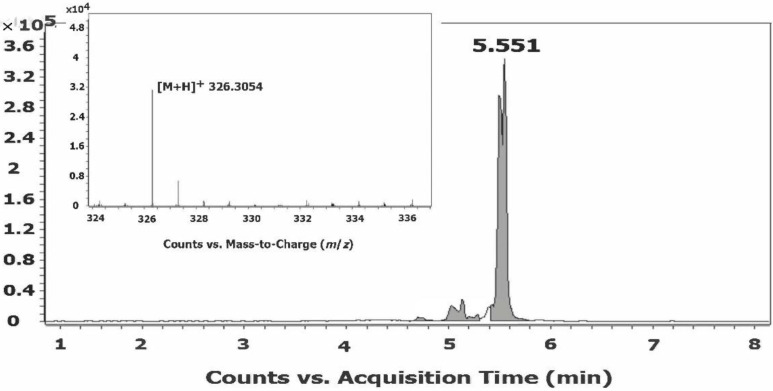
Identification of *N*-oleoylethanolamine in an extract prepared from *T. castaneum* by LC/QTOF-MS. The EIC peak and mass spectrum (inset) of endogenous *N*-oleoylethanolamine matched those of the *N*-oleoylethanolamine standard (Supplementary Materials Figure S9). These data are representative of the data collected for each long-chain fatty acid amide identified from *T. castaneum*.

*T. castaneum* is a significant worldwide pest for stored agricultural products [41]. Our characterization of the fatty acid amidome for *T. castaneum* could serve as a basis for the development of new insecticides to control this pest, by targeting the enzymes responsible for their metabolism or the receptors involved in their biological function. The extracted-ion chromatogram (EIC) and mass spectrum of a standard, *N*-oleoylethanolamine (Supplementary Materials Figure S9), matched those of endogenous *N*-oleoylethanolamine identified in extracts from *T. castaneum* (Figure 2). LC–MS/MS analysis of the *N*-oleoylethanolamine parent ion from the *T. castaneum* extract yielded product ion scans with the expected fragment ion for an *N*-acylethanolamine, 62.1 (Supplementary Materials Figure S10). These data are representative of the data collected for each fatty acid amide identified in the *T. castaneum* (Table 4).

### 2.5. Future Directions

Because the theme of this special issue of *Molecules* is the “Synthesis, Quantification and NMR Characterization of Bioactive Compounds,” we have refrained from detailed comparisons of the fatty acid amidomes reported herein (Table 1, Table 2, Table 3 and Table 4) or the cellular functions of the individual fatty acid amides. The functions of most of the fatty acid amides are unknown or unclear [5,35]. Anandamide [4,5,42], oleamide [43], and *N*-palmitoylethanolamine [44] are signaling molecules suggesting that all the fatty acid amides have a cellular signaling function. The question of function must be answered or the fatty acid amides will be relegated to brief mentions in future reviews of cell signaling lipids. The question of function is challenging to answer. One possible solution would be an approach akin to activity-based proteomic profiling based upon the synthesis of reactive fatty acid amide analogs that would facilitate the identification of receptors and proteins that bind specifically and with high affinity to individual members of the fatty acid amide family [45,46].

Another area of future research is the proteins and enzymes involved in fatty acid amide metabolism and transport. Pathways for the biosynthesis, degradation, and modification of anandamide and the other *N*-acylethanolamines are known [47,48,49]. Enzymes catalyzing the formation of other classes of fatty acid amides have been described, including the *N*-acylglycines [16,50,51], the PFAMs [14,16], and the *N*-acyl-arylalkylamides [17,19]. One conundrum is a biosynthetic route for the *N*-acylamino acids except for the *N*-acylglycines. Recall that Tortoriello et al. [39], found a series of *N*-acylamino acids are produced by *D. melanogaster* larvae (Table 2). The enzymes known to catalyze *N*-acylglycine formation will not accept other amino acids as substrates [52] and the enzymes known to catalyze *N*-acyl-arylalkylamides formation will not accept amino acids as substrates [17,19]. Questions remain about fatty acid amide degradation because it has not been fully established if all the fatty acid amide classes are substrates for the fatty acid amide hydrolases. Furthermore, it is unclear if the fatty acyl hydroxylation reactions identified for anandamide [49,53] would occur for the other fatty acid amides. Finally, there is the question of transport. The fatty acid amides are of limited aqueous solubility and the issue of fatty acid amide transporters has not been completely resolved.

## 3. Materials and Methods

### 3.1. General Information

^1^H-NMR (400 and 600 MHz) and ^13^C-NMR (151 MHz) spectra were recorded at 25 °C on Bruker 600 MHz and 400 MHz nuclear magnetic resonance instruments in DMSO-D_6_ or CDCl_3_. (Supplementary Materials Figures S1–S8). ESI mass spectra were measured on an Agilent Technologies LC-MS QTOF 6540 mass spectrometer (Agilent Technologies Japan, Ltd., Tokyo, Japan). All chromatographic separations were accomplished with Silica Gel. Thin-layer chromatography (TLC) was performed with pre-coated TLC plates UV_254_. Spectrophotometric analyses were performed on a Cary 300 Bio UV-Visible spectrophotometer. All the reagents, cell culture supplies, and insect chow were of the highest quality available form commercial suppliers and are were used without further purification, unless otherwise noted. Most of the reagents were used without further purification unless otherwise specified.

### 3.2. N-(2-Hydroxyethyl)Oleamide (FA-1, N-Oleoylethanolamine)

To acyl chloride (5.0 mmol, 1.945 mL) in 15 mL of dichloromethane was added dropwise of ethanolamine (7.5 mmol, 0.46 mL). 1.40 mL triethylamine dissolved in 15 mL of dichloromethane was subsequently added. The reaction mixture was stirred for 1 h before evaporated to dryness. The light brown crude was obtained. The crude was purified using silica gel column (hexane to methanol: DCM = 1:2) to afford white solid (1.62 g, yield 90%). ^1^H NMR (400 MHz, CDCl_3_) δ 6.02 (s,1H), 5.36–5.27 (m, 2H), 3.69 (t, J = 4.8 Hz, 2H), 3.42 (dt, J = 4.8, 5.2 Hz, 2H), 2.73 (s, 1H), 2.18 (t, J = 7.7 Hz, 2H), 1.99–1.97 (m, 4H), 1.63–1.59 (m, 2H), 1.27–1.24 (20H), 0.85 (t, J = 6.8 Hz, 3H). ^13^C NMR (151 MHz, CDCl_3_) δ 174.63, 130.03, 129.72, 62.37, 42.43, 36.68, 31.91, 29.77, 29.72, 29.54, 29.33, 29.28, 29.15, 27.23, 27.18, 25.74, 22.69, 14.13. HRMS (ESI) = *m*/*z* [M]^+^ calculated for C_20_H_39_NO_2_:325.2981 Found: 325.2991 [22,54].

### 3.3. N-Oleoylglycine (FA-2)

Oleoyl chloride (13.29 mmol, 5.17 mL (85%)) in absolute THF (20 mL) was added slowly over a 30 min period, with a dropping funnel, in an aqueous NaOH (2 M) solution (40 mL) of amino acid (19.94 mmol, 1.5 g), which was immersed in ice bath. The solution, along with the generated white precipitate, was stirred in an ice bath for an additional hour, and then at room temperature for 12 hr. Subsequently, water (10 mL) was added to dissolve the precipitate, and then aq. HCl (3 M, 20 mL) was added to reduce the pH of the solution to <2. The generated white precipitate was filtered, rinsed with water, and subsequently dried in vacuo. The crude was purified using silica gel column (hexane to methanol: DCM = 5%:100%) to afford white solid (2.5 g, yield 55.4%). ^1^H NMR (600 MHz, DMSO) δ 8.08 (t, J = 5.9 Hz, 1H), 5.33–5.32 (m, 2H), 3.71 (d, J = 6.0 Hz, 2H), 2.09 (t, J = 7.4 Hz, 2H), 2.00–1.96 (m, 4H), 1.49–1.24 (20H), 0.85 (t, 7.0 Hz, 3H). ^13^C NMR (151 MHz, DMSO) δ 172.98, 171.93, 130.08, 40.96, 35.51, 31.77, 29.59, 29.33, 29.19, 29.09, 27.09, 27.05, 25.66, 22.59, 14.42. HRMS (ESI) = *m*/*z* [M]^+^ calculated for C_20_H_37_NO_3_: 339.2773 Found: 339.2786 [23,55].

### 3.4. Palmitamide (FA-3)

Add amine (2.2 mL, 14.3 mmol), acyl chloride (4.4 mL, 14.3 mmol), THF (70.0 mL) and Et_3_N (4.0 mL, 28.6 mmol, 2.0 equivalents) to a round bottom flask open to air. Heat the reaction mixture in an oil bath at 65 °C for 12 hr. Add H_2_O (70.0 mL) to the reaction mixture. Extract the reaction mixture with ethyl acetate. Combine the organic layers. Remove the organic solvent in vacuo. White solid was obtained. The crude was purified using silica gel column (hexane to methanol: DCM = 5.0%:100%) to afford white solid (1.2 g, yield 32.8%). ^1^H NMR (600 MHz, CDCl_3_) δ 5.50–5.45 (bs, 2H), 2.24 (t, 7.7 Hz, 2H), 1.68–1.63 (m, 2H), 1.36–1.27 (24H), 0.89 (t, 7.1 Hz, 3H). ^13^C NMR (151 MHz, CDCl_3_) δ 175.95, 35.97, 31.93, 29.69, 29.66, 29.62, 29.48, 29.37, 29.35, 29.24, 25.55, 22.70, 14.13. HRMS (ESI) = *m*/*z* [M]^+^ calculated for C_16_H_33_NO: 255.2562 Found: 255.2570 [24,56].

### 3.5. N-Palmitoylglycine (FA-4)

Palmitoyl chloride (14.3 mmol, 4.4 mL in absolute THF (20 mL)) was added slowly over a 30 min period, with a dropping funnel, in an aqueous NaOH (2 M) solution (40 mL) of amino acid (14.3 mmol, 1.07 g), which was immersed in an ice bath. The solution, along with the generated white precipitate, was stirred in an ice bath for an additional hour, and then at room temperature for 12 hr. Subsequently, water (10 mL) was added to dissolve the precipitate, and then aq. HCl (3M, 20 mL) was added to reduce the pH of the solution to <2. The generated white precipitate was filtered, rinsed with water, and, subsequently, dried in vacuo. A white solid was obtained. The crude was purified using silica gel column (hexane to methanol: DCM = 5%:100%) to afford white solid (1.3 g, yield 29%). ^1^H NMR (600 MHz, DMSO) δ 8.08 (t, 5.7 Hz, 1H), 3.70 (d, 5.9 Hz, 2H), 2.09 (t, 7.4 Hz, 2H), 1.49–1.46 (m, 2H), 1.28–1.24 (24H), 0.85 (t, 7.0 Hz, 3H). ^13^C NMR (151 MHz, DMSO) δ 173.02, 40.99, 35.53, 31.77, 29.52, 29.28, 29.18, 29.08, 25.65, 22.57, 14.43. HRMS (ESI) = *m*/*z* [M]^+^ calculated for C_18_H_35_NO_3_:313.2617 Found: 313.2625 [23,57].

### 3.6. Cells and Cell Culture

Mouse neuroblastoma N_18_TG_2_ cells were from DSMZ (Deutsche Sammlung von Mikrooganism und Zellkuturen GmBH) and the sheep choroid plexus (SCP) cells were from the American Type Culture Collection. The cells were grown and harvested as described [15,16].

### 3.7. Insects

*Drosophila melanogaster* (Oregon R) were purchased from Carolina Biological and were reared on 4–24 Instant Medium. The flies were collected after a 5-day incubation at room temperature by immobilization on ice, flash-frozen in liquid N_2_, and the frozen flies were shaken vigorously to detach the head from the thorax–abdomen. The heads were separated from thorax–abdomens by sifting through a wire mesh and stored at −80 °C until analysis of fatty acid amidome analysis [18].

*Bombyx mori* eggs (domesticated silkworm) were from Carolina Biological and were reared on Silkworm Artificial Dry Diet at room temperature. The larvae were grown until the fourth instar, three molts after the original hatch. The fourth instar larvae, Bmi4, were selected to be identical in size and development, were flash-frozen with liquid N_2_, and stored at −80 °C until analysis of the fatty acid amidome [19].

*Tribolium castaneum* (red flour beetle) were a gift from Dr. Susan J. Brown (Department of Biology, Kansas State University) and were maintained at 35 °C in a growth media that consisted of 9.5 g of pre-sifted organic whole wheat flour and 0.5 g of brewer’s yeast. The organic whole wheat flour was obtained at a local food store. Adult beetles were removed after egg-laying and larvae development, flash-frozen in liquid N_2_, and stored at −80 °C until analysis of the fatty acid amidome [21].

Approximately 200 adult female worker bees (*A. mellifera*) from a local colony were a gift from the USF Botanical Gardens in cooperation with Dr. Brent Weisman (Department of Anthropology, University of South Florida). The donated bees had been immobilized on dry ice. After the immobilized bees were delivered, they were flash-frozen in liquid N_2_ until thoroughly frozen. The frozen bees were placed in a clean, tightly sealed container and shaken vigorously to separate the head, thorax, and abdomen. The legs, wing, antennae, loose pollen, and other particulate matter settled to collected on the bottom of the container enabling easy separation for the desired segments. The head, thorax, and abdomen were stored separated at −80 °C until analysis of the fatty acid amidome [21].

### 3.8. Characterization of the Fatty Acid Amidome

The methods we use to extract the fatty acid amides from the desired biological source and to characterize the fatty acid amidome by LC/QTOF-MS were detailed in Jeffries et al. [20]. Our extraction method is based on the procedure from Sultana and Johnson [58]. The individual fatty acid amides identified from the extracts were quantified using standard curves constructed using the appropriate pure standard, either obtained by chemical synthesis or from a commercial supplier.

## 4. Conclusions

We have described the synthesis and characterization of fatty acid amides, which were then employed as standards in our LC/QTOF-MS to characterize and quantify fatty acid amides from cultured mouse N_18_TG_2_ and SCP cells and in every insect we examined. The fatty acid amides are an intriguing family of biologically occurring lipids that likely are cell signaling. There remain many unanswered questions about the fatty acid amides. The answers to these questions should provide new insights into both vertebrate and invertebrate biology and new targets for the treatment of human disease and the control of insect pests.

## Figures and Tables

**Table 1:** Fatty acid amides produced by the mouse neuroblastoma N_18_TG_2_ cells and sheep choroid plexus (SCP) cells ^1^.

Fatty Acid Amide	N_18_TG_2_ Cells	SCP Cells ^5^
**➤ *N*-Acylglycines**		
*N*-Palmitoylglycine ^2^	+	-
*N*-Oleoylglycine ^2^	++	-
**➤ *N*-Acylethanolamines**		
*N*-Palmitoylethanolamine ^3^	+	-
*N*-Oleoylethanolamine ^2,3^	+	-
*N*-Stearoylethanolamine ^3^	+	-
*N*-Linoleoylethanolamine ^3^	+	-
Anandamide ^3^	+	-
**➤ Primary Fatty Acid Amides**		
Palmitoleamide ^2^	+	++
Palmitamde ^2^	+++	+++
Oleamide ^2,4^	+++	+++
Linoleamide ^2^	++	++
**➤ *N*-Acyldopamines**		
*N*-Palmitoyldopamine ^2^	++	-
*N*-Oleoyldopamine ^2^	+	-
*N*-Archidonoyldopamine ^2^	*+*	*-*

^1^ The relative abundance of each fatty acid amide in N_18_TG_2_ cells or SCP cells is represented by the number of plus signs (+), with + representing low abundance (10–100 pmoles/10^7^ cells), ++ representing a middle level of abundance (100–500 pmoles/10^7^ cells), and +++ representing high abundance (>500 pmoles/10^7^ cells). Please see the indicated references for the levels measured. The dash (-) indicates that no measurements were made in the SCP cells for the indicated fatty acid amide. ^2^ Data for the N_18_TG_2_ cells from Jeffries et al. [16]. ^3^ Data for the N_18_TG_2_ cells from Di Marzo et al. [26]. ^4^ Data for the N_18_TG_2_ cells from Bisogno et al. [25]. ^5^ Data for the SCP cells from Farrell et al. [15].

**Table 2:** Fatty acid amides produced by the *Drosophila melanogaster* ^1^.

Fatty Acid Amide	Larvae ^2,3^	Head ^4^	Thorax-Abdomen ^4^
**➤ *N*-Acylalanine**			
*N*-Palmitoylalanine	detected	-	-
*N*-Stearoylalanine	detected	-	-
*N*-Oleoylalanine	detected	-	-
*N*-Linoleoylalanine	detected	-	-
**➤ *N*-Acyl-γ-aminobutyrates**			
*N*-Oleoyl-γ-aminobutyrate	detected	-	-
*N*-Linoleoyl-γ-aminobutyrate	detected	-	-
**➤ *N*-Acylglycines**			
*N*-Palmitoylglycine	+	+	+
*N*-Stearoylglycine	detected	not detected	not detected
*N*-Oleoylglycine	+	++	+
*N*-Linoleoylglycine	+	++	+
*N*-Arachidonoylglycine ^5^	+, not detected	not detected	not detected
**➤ *N*-Acylleucines**			
*N*-Palmitoylleucine	detected	-	-
*N*-Stearoylleucine	detected	-	-
*N*-Oleoylleucine	detected	-	-
*N*-Linoleoylleucine	detected	-	-
**➤ *N*-Acylmethionines**			
*N*-Palmitoylmethionine	detected	-	-
*N*-Oleoylmethionine	detected	-	-
*N*-Linoleoylmethionine	detected	-	-
**➤ *N*-Acylphenylalanines**			
*N*-Palmitoylphenylalanine	detected	-	-
*N*-Stearoylphenylalanine	detected	-	-
*N*-Oleoylphenylalanine	detected	-	-
*N*-Linoleoylphenylalanine	detected	-	-
**➤ *N*-Acylprolines**			
*N*-Palmitoylproline	detected	-	-
*N*-Stearoylproline	detected	-	-
*N*-Oleoylproline	detected	-	-
*N*-Linoleoylproline	detected	-	-
**➤ *N*-Acylserines**			
*N*-Palmitoylserine	detected	-	-
*N*-Stearoylserine	detected	-	-
*N*-Oleoylserine	detected	-	-
*N*-Linoleoylserine	detected	-	-
**➤ *N*-Acyltryptophans**			
*N*-Palmitoyltryptophan	detected	-	-
*N*-Stearoyltryptophan	detected	-	-
*N*-Oleoyltryptophan	detected	-	-
*N*-Linoleoyltryptophan	detected	-	-
**➤ *N*-Acyltyrosines**			
*N*-Palmitoyltyrosine	detected	-	-
*N*-Stearoyltyrosine	detected	-	-
*N*-Oleoyltyrosine	detected	-	-
*N*-Linoleoyltyrosine	detected	-	-
**➤ *N*-Acylvalines**			
*N*-Palmitoylvaline	detected	-	-
*N*-Stearoylvaline	detected	-	-
*N*-Oleoylvaline	detected	-	-
*N*-Linoleoylvaline	detected	-	-
**➤ *N*-Acylethanolamines**			
*N*-Palmitoylethanolamine	detected	not detected	not detected
*N*-Stearoylethanolamine	detected	not detected	not detected
*N*-Oleoylethanolamine	+	+	+
*N*-Linoleoylethanolamine	detected	not detected	not detected
Anandamide ^5^	+, not detected	+	+
**➤ *N*-Acyldopamines**			
*N*-Palmitoyldopamine	+	+	+
*N*-Oleoyldopamine	+	not detected	+
*N*-Arachidonoyldopamne	+	+	not detected
**➤ *N*-Acylserotonins**			
*N*-Palmitoylserotonin	+	-	-
*N*-Oleoylserotonin	+	-	-
*N*-Arachidonoylserotonin	+	-	-
**➤ Primary Fatty Acid Amides**			
Palmitamide	+	+	not detected
Palmitoleamide	+	+	not detected
Oleamide	+	++	not detected
Linoleamide	+	+	not detected

^1^ The relative abundance of each fatty acid amide from the larvae, head, or thorax-abdomen of *D. melanogaster* is represented by the number of plus signs (+), with + representing low abundance (10–100 pmoles/g), ++ representing a middle level of abundance (100–500 pmoles/g), and +++ representing high abundance (>500 pmoles/g). Please see the indicated references for the levels measured, reported as pmoles/(g of tissue). The dash (-) indicates that no measurements were made for the indicated fatty acid amide from *D. melanogaster*. ^2^ Data for *D. melanogaster* larvae from Suarez and Merkler [38]. ^3^ Data for *D. melanogaster* larvae from Tortoriello et al. [39]. ^4^ Data for *D. melanogaster* head and thorax-abdomen from Jeffries et al. [18]. ^5^ Tortoriello et al. [39] report not detected and we identified low levels in larvae (Supplementary Materials Table S1).

**Table 3:** Fatty acid amides produced by *Apis mellifera* ^1,2^.

Fatty Acid Amide	Head	Thorax	Abdomen
**➤ *N*-Acylglycines**			
*N*-Palmitoylglycine	+	+	++
*N*-Oleoylglycine	+	+	+
*N*-Arachidonoylglycine	+	detected ^3^	
**➤ *N*-Acylethanolamines**			
*N*-Oleoylethanolamine	+	+	+++
Anandamide	+	+	detected ^3^
**➤ *N*-Acyldopamines**			
*N*-Palmitoyldopamine	+	+	++
*N*-Oleoyldopamine	+	detected ^3^	detected ^3^
*N*-Arachidonoyldopamine	detected ^3^	detected ^3^	detected ^3^
**➤ *N*-Acylserotonin**			
*N*-Oleoylserotonin	+	+	++
**➤ Primary Fatty Acid Amides**			
Palmitamide	++	+	+
Palmitoleamide	++	+++	+++
Oleamide	++	+++	++
Linoleamide	+	+	++

^1^ The relative abundance of each fatty acid amide from the head, thorax, and abdomen of *A. mellifera* is represented by the number of plus signs (+), with + representing low abundance (10–100 pmoles/g), ++ representing a middle level of abundance (100–500 pmoles/g), and +++ representing high abundance (>500 pmoles/g). Please see the indicated references for the levels measured, reported as pmoles/(g of tissue). ^2^ Data from Mitchell [21]. ^3^ Detected, the indicated fatty acid amide was detected, but could not be reliably quantified.

**Table 4:** Fatty acid amides produced by *Tribolium castaneum* ^1,2^ and Bombyx mori ^1,3^.

Fatty Acid Amide	*T. Castaneum*	*B. Mori*
**➤ *N*-Acylglycines**		
*N*-Palmitoylglycine	+	not detected
*N*-Oleoylglycine	+	+
*N*-Arachidonoylglycine	+	not detected
**➤ *N*-Acylethanolamines**		
*N*-Oleoylethanolamine	+	+
Anandamide	+	not detected
**➤ *N*-Acyldopamines**		
*N*-Palmitoyldopamine	+	not detected
*N*-Oleoyldopamine	+	+
*N*-Arachidonoyldopamine	+	not detected
**➤ *N*-Acylserotonins**		
*N*-Palmitoylserotonin	not detected	+
*N*-Stearoylserotonin	not detected	+
*N*-Oleoylserotonin	detected ^4^	+
**➤ Primary Fatty Acid Amides**		
Palmitamide	+	+
Palmitoleamide	++	+
Oleamide	+	+
Linoleamide	detected ^4^	+

^1^ The relative abundance of each fatty acid amide from *T. castaneum* or *B. mori* is represented by the number of plus signs (+), with + representing low abundance (10–100 pmoles/g) and ++ representing a middle level of abundance (100–500 pmoles/g). Please see the indicated references for the levels measured, reported as pmoles/(g of tissue). ^2^ Data for *T. castaneum* (whole body) from Mitchell [21]. ^3^ Data for the 4th instar *B. mori* from Anderson et al. [19]. ^4^ Detected, the indicated fatty acid amide was detected, but could not be reliably quantified.

## Data Availability

All data are contained within the article, in the experimental section and the supplementary material.

## Supplementary Materials

The following are available online. Table S1: Identification and Quantification of Fatty Acid Amides Produced by *Drosophila melanogaster Larvae* by LC/QTOF-MS. Figure S1: ^1^H NMR (600 MHz, CDCl_3_) of **FA-1**, the inset shows multiplicity of the signals. Figure S2: ^13^CNMR (151 MHz, CDCl_3_) of **FA-1**, the inset shows clusterd signals. Figure S3: ^1^H NMR (600 MHz, DMSO) of **FA-2**, the inset shows multiplicity of the signals. Figure S4: ^13^C NMR (151 MHz, DMSO) of **FA-2**, the inset shows clusterd signals. Figure S5: ^1^H NMR (600 MHz, CDCl_3_) of **FA-3**, the inset shows multiplicity of the signals. Figure S6: ^13^C NMR (151 MHz, CDCl_3_) of **FA-3**, the inset shows clusterd signals. Figure S7: ^1^H NMR (600 MHz, DMSO) of **FA-4**, the inset shows multiplicity of the signals. Figure S8: ^13^C NMR (151 MHz, DMSO) of **FA-4**, the inset shows clusterd signals. Figure S9: The EIC peak and mass spectrum of the *N*-oleoylethanolamine standard. Figure S10: Targeted MS/MS spectra of endogenous *N*-oleoylethanolamine in the *T. castaneum* extract at 20 eV collision energy displaying expected primary fragment ion, m/z 62.0602.

## Sample Availability

Samples of the compounds are available from the authors.

## References

[1] Bradshaw H.B., Leishman E. (2017). Lipidomics: A Corrective Lens of Enzyme Mopia. Methods Enzymol..

[2] Bradshaw H.B., Walker J.M. (2005). The Expanding Field of Cannabimimetic and Related Mediators. Br. J. Pharmacol..

[3] Waluk D.P., Battistini M.R., Dempsey D.R., Farrell E.K., Jeffries K.A., Mitchell P., Hernandez L.W., McBride J.C., Merkler D.J., Hunt M.C., Watson R.R., DeMeester F. (2014). Mammalian Fatty Acid Amides of the Brain and CNS. Omega-3 Fatty Acids in Brain and Neurological Health.

[4] Maccarrone M., Bab I., Bíro T., Cabral G.A., Dey S.K., Di Marzo V., Konje J.C., Kunos G., Mechoulam R., Pacher P. (2015). Endocannabinoid Signaling at the Periphery: 50 Years after THC. Trends Pharmacol. Sci..

[5] Iannotti F.A., Di Marzo V., Petrosino S. (2016). Endocannabinoids and Endocannabinoid-Related Mediators: Targets, Metabolism and Role in Neurological Disorders. Prog. Lipid Res..

[6] Battista N., Bari M., Bisogno T. (2019). *N*-Acyl Amino Acids: Metabolism, Molecular Targets, and Role in Biological Processes. Biomolecules.

[7] Llop C., Manrique A., Navarro R., Mijangos C., Reinecke H. (2011). Control of the Migration Behavior of Slip Agents in Polyolefin-Based Films. Polym. Eng. Sci..

[8] Hawthorne J.N. (1975). A Note on the Life of J.L.W. Thudichum (1829–1901). Biochem. Soc. Trans..

[9] Kuehl F.A., Jacob T.A., Ganley O.H., Ormond R.E., Meisinger M.A.P. (1957). The Identification of N-(2-Hydroxyethyl)-Palmitamide as a Naturally Occurring Anti-Inflammatory Agent. J. Am. Chem. Soc..

[10] Arafat E.S., Trimble J.W., Andersen R.N., Dass C., Desiderio D.M. (1989). Identification of Fatty Acid Amides in Human Plasma. Life Sci..

[11] Devane W.A., Hanuš L., Breuer A., Pertwee R.G., Stevenson L.A., Griffin G., Gibson D., Mandelbaum A., Etinger A., Mechoulam R. (1992). Isolation and Structure of a Brain Constituent That Binds to the Cannabinoid Receptor. Science.

[12] Alborn H.T., Turlings T.C.J., Jones T.H., Stenhagen J.H., Loughrin J.H., Tumlinson J.H. (1997). An Elicitor of Plant Volatiles from Beet Armyworm Oral Secretion. Science.

[13] Cravatt B.F., Prospero-Garcia O., Siuzdak G., Gilula N.B., Henriksen S.J., Boger D.L., Lerner R.A. (1995). Chemical Characterization of a Family of Brain Lipids that Induce Sleep. Science.

[14] Merkler D.J., Merkler K.A., Stern W., Fleming F.F. (1996). Fatty Acid Amide Biosynthesis: A Possible New Role for Peptidylglycine α-Amidating Enzyme and Acyl-CoA:Glycine *N*-Acyltransferase. Arch. Biochem. Biophys..

[15] Farrell E.K., Chen Y., Barazanji M., Jeffries K.A., Cameroamortegui F., Merkler D.J. (2012). Primary Fatty Acid Amide Metabolism: Conversion of Fatty Acids and an Ethanolamine in N_18_TG_2_ and SCP Cells. J. Lipid Res..

[16] Jeffries K.A., Dempsey D.R., Farrell E.K., Anderson R.L., Garbade G.J., Gurina T.S., Gruhonjic I., Gunderson C.A., Merkler D.J. (2016). Glycine *N*-Acyltransferase-like 3 is Responsible for Long-chain *N*-Acylglycine Formation in N_18_TG_2_ Cells. J. Lipid Res..

[17] Dempsey D.R., Jeffries K.A., Anderson R.L., Carpenter A.-M., Rodriquez Ospina S., Merkler D.J. (2014). Identification of an Arylalkylamine *N*-Acyltransferase from *Drosophila melanogaster* that Catalyzes the Formation of Long-chain *N*-Acylserotonins. FEBS Lett..

[18] Jeffries K.A., Dempsey D.R., Behari A.L., Anderson R.L., Merkler D.J. (2014). *Drosophila melanogaster* as a Model System to Study Long-chain Fatty Acid Amide Metabolism. FEBS Lett..

[19] Anderson R.L., Battistini M.R., Wallis D.J., Shoji C., O’Flynn B.G., Dillashaw J.E., Merkler D.J. (2018). *Bm*-iAANAT and Its Potential Role in Fatty Acid Amide Biosynthesis in *Bombyx mori*. Prostaglandins Leukot. Essent. Fatty Acids.

[20] Jeffries K.A., Farrell E.K., Anderson R.L., Suarez G., Osborne A.J.G., Heide M.K., Merkler D.J., Wood P.L. (2021). Characterization and Quantification of the Fatty Acid Amidome. Metabolomics.

[21] Mitchell P.R. (2015). The Detection and Quantitative Analysis of Endocannabinoids and Endogenous Fatty Acid Amides in *Apis mellifera* and *Tribolium castneum*. Master’s Thesis.

[22] Morales-Sanfrutos J., Megia-Fernandez A., Hernandez-Mateo F., Giron-Gonzalez D., Salto-Gonzalez R., Santoyo-Gonzalez F. (2011). Alkyl Sulfonyl Derivatized PAMAM-G2 Dendrimers as Nonviral Gene Delivery Vectors with Improved Transfection Efficiencies. Org. Biomol. Chem..

[23] Ohsedo Y., Oono M., Saruhashi K., Watanabe H., Miyamoto N. (2017). Thixotropic Stiff Hydrogels from a New Class of Oleoyl-D-Glutamine-Based Low-Molecular-Weight Gelators. RSC Adv..

[24] Ji Y.-F., Yan H., Jiang Q.-B. (2015). Effective Nitration of Anilides and Acrylamides by *tert*-Butyl Nitrite. Eur. J. Org. Chem..

[25] Bisogno T., Sepe N., De Petrocellis L., Mechoulam R., Di Marzo V. (1997). The Sleep Inducing Factor Oleamide is Produced by Mouse Neuroblastoma Cells. Biochem. Biophys. Res. Commun..

[26] Di Marzo V., De Petrocellis L., Sepe N., Buono A. (1996). Biosynthesis of Anandamide and Related Acylethanolamides in Mouse J774 Macrophages and N_18_ Neuroblastoma Cells. Biochem. J..

[27] Ritenour-Rodgers K.J., Driscoll W.J., Merkler K.A., Merkler D.J., Mueller G.P. (2000). Induction of Peptidylglycine α-Amidating Monooxygenase in N_18_TG_2_ Cells: A Model for Studying Oleamide Biosynthesis. Biochem. Biophys. Res. Commun..

[28] Merkler D.J., Chew G.H., Gee A.J., Merkler K.A., Sorondo J.-P.O., Johnson M.E. (2004). Oleic Acid Derived Metabolites on Mouse Neuroblastoma N_18_TG_2_ Cells. Biochemistry.

[29] Lun M.P., Monuki E.S., Lehtinen M.K. (2015). Development and Functions of the Choroid Plexus-Cerebrospinal Fluid System. Nat. Rev. Neurosci..

[30] Gee P., Rhodes C.H., Fricker L.D., Angeletti R.H. (1993). Expression of Neuropeptide Processing Enzymes and Neurosecretory Proteins in Ependyma and Choroid Plexus Epithelium. Brain Res..

[31] Tsukamoto T., Noguchi M., Kayama H., Watanabe T., Asohi T., Yamamoto T. (1995). Increased Peptidylglycine α-Amidating Monooxygease Activity in Cerebrospinal Fluid of Patients with Multiple Schlerosis. Intern. Med..

[32] Gulaya N.M., Volkov G.L., Klimashevsky V.M., Glovseeva N.N., Melnik A.A. (1989). Changes in Lipid Composition of Neuroblastoma C1300 N18 Cell During Differentiation. Neuroscience.

[33] Marchioni C., de Souza I.D., Junior V.R.A., de Souza Crippa J.A., Tumas V., Queiroz M.E.C. (2018). Recent Advances in LC-MS/MS Methods to Determine Endocannabinoids in Biological Samples: Application in Neurodegenerative Diseases. Anal. Chim. Acta.

[34] Bradshaw H.B., Rimmerman N., Hu S.S.-J., Burstein S., Walker J.M. (2009). Novel Endogenous *N*-Acyl Glycines: Identification and Characterization. Vitam. Horm..

[35] Bradshaw H.B., Lee S.H., McHugh D. (2009). Orphan Endogenous Lipids and Orphan GPCRs: A Good Match. Prostaglandins Other Lipid Mediat..

[36] O’Flynn B.G., Suarez G., Hawley A.J., Merkler D.J. (2018). Insect Arylalkylamine *N*-Acyltransferases: Mechanism and Role in Fatty Acid Amide Biosynthesis. Front. Mol. Biosci..

[37] McParland J., Di Marzo V., De Petrocellis L., Mercer A., Glass M. (2001). Cannabinoid Receptors are Absent in Insects. J. Comp. Neurol..

[38] Suarez G., Merkler D.J. (2021). University of South Florida, Tampa, FL, USA.

[39] Tortoriello G., Rhodes B.P., Takacs S.M., Stuart J.M., Basnet A., Raboune S., Widlanski T.S., Doherty P., Harkany T., Bradshaw H.B. (2013). Target Lipidomic in *Drosophila melanogaster* Identified Novel 2-Monoacylglycerols and *N*-Acyl Amides. PLoS ONE.

[40] Meng X., Zhu F., Chen K. (2017). Silkworm: A Promising Model Organism in Life Science. J. Insect Sci..

[41] Dissanayaka D.M.S.K., Sammani A.M.P., Wijayaratne L.K.W. (2020). Response of Different Population Sizes to Traps and Effect of Spinosad on the Trap Catch and Progeny Adult Emergence in *Tribolium castaneum* (Herbst) (Coleoptera: Tenebrionidae). J. Stored Prod. Res..

[42] Lu H.-C., Mackie K. (2016). An Introduction to the Endogenous Cannabinoid System. Biol. Psychiatry.

[43] Prospéro-García O., Amancio-Belmont O., Meléndez A.L.B., Ruiz-Contreras A.E., Méndez-Díaz M. (2016). Endocannabinoids and Sleep. Neurosci. Biobehav. Rev..

[44] Alhouayek M., Muccioli G.G. (2014). Harnessing the Anti-Inflammatory Potential of Palmitoylethanolamide. Drug Discov. Today.

[45] Niphakis M.J., Lum K.M., Cognetta A.B., Correia B.E., Ichu T.-A., Olucha J., Brown S.J., Kundu S., Piscitelli F., Rosen H. (2015). A Global Map of Lipid-Binding Proteins ant Their Ligandability in Cells. Cell.

[46] Merkler D.J., Leahy J.W. (2018). Binding-Based Proteomic Profiling and the Fatty Acid Amides. Trends Res..

[47] Sun Y.X., Tsuboi K., Okamoto Y., Tonai T., Murakami M., Kudo I., Ueda N. (2004). Biosynthesis of Anandamide and *N*-Palmitoylethanolamine by Sequential Actions of Phospholipase A_2_ and Lysophospholipase D. Biochem. J..

[48] Ueda N., Tsuboi K., Uyama T. (2010). Enzymological Studies on the Biosynthesis of *N*-Acylethanolamines. Biochim. Biophys. Acta.

[49] Maccarrone M. (2017). Metabolism of the Endocannabinoid Anandamide: Open Questions after 25 Years. Front. Mol. Neurosci..

[50] Waluk D.P., Schultz N., Hunt M.C. (2010). Identification of Glycine *N*-Acyltransferase-like 2 (GLYATL2) as a Transferase that Produces *N*-Acyl Glycines in Humans. FASEB J..

[51] Aneetha H., O’Dell D.K., Tan B., Walker J.M., Hurley T.D. (2009). Alcohol Dehydrogenase-Catalyzed In Vitro Oxidation of Anandamide to *N*-Arachidonoyl Glycine, a Lipid Mediator: Synthesis of *N*-Acyl Glycinals. Bioorg. Med. Chem. Lett..

[52] Dempsey D.R., Bond J.D., Carpenter A.-M., Ospina S.R., Merkler D.J. (2014). Expression, Purification, and Characterization of Mouse Glycine *N*-Acyltransferase in *Esherichia coli*. Protein Expr. Purif..

[53] Rouzer C.A., Marnett L.J. (2011). Endocannabinoid Oxygenation by Cyclooxygenases, Lipoxygenases, and Cytochromes P450: Cross-Talk between the Eicosanoid and Endocannabinoid Signaling Pathways. Chem. Rev..

[54] Plastina P., Meijerink J., Vincken J.-P., Gruppen H., Witkamp R., Gabriele B. (2009). Selective Synthesis of Unsaturated N-Acylethanolamines by Lipase-Catalyzed N-Acylation of Ethanolamine with Unsaturated Fatty Acids. Lett. Org. Chem..

[55] Goujard L., Figueroa M.C., Villeneuve P. (2004). Chemo-Enzymatic Synthesis of *N*-Arachidonoyl Glycine. Biotechnol. Lett..

[56] Vandevoorde S., Jonsson K.-O., Fowler C.J., Lambert D.M. (2003). Modifications of the Ethanolamine Head in *N*-Palmitoylethanolamine Synthesis and Evaluation of New Agents Interfering with the Metabolism of Anandamide. J. Med. Chem..

[57] Dang H.T., Kang G.J., Yoo E.S., Hong J., Choi J.S., Kim H.S., Chung H.Y., Jung J.H. (2011). Evaluation of Endogenous Fatty Acid Amides with Their Synthetic Analogues as Potential Anti-inflammatory Leads. Bioorg. Med. Chem..

[58] Sultana T., Johnson M.E. (2006). Sample Preparation and Gas Chromatography of Primary Fatty Acid Amides. J. Chromatogr. A.

