# Supplementary material for: Synthesis, Quantification, and Characterization of Fatty Acid Amides from In Vitro and In Vivo Sources
Source: Molecules. 2021 Apr 27;26(9):2543. doi: 10.3390/molecules26092543 (PMC8123904; doi:10.3390/molecules26092543)
Supplement: Supplementary file 1 [file molecules-26-02543-s001.zip › molecules-1192270-supplementary.pdf]

Supporting Information

Synthesis, Quantification, and Characterization of Fatty Acid Amides from *In Vitro* and *In Vivo* Sources

Ruidong Ni, Suzeeta Bhandari, Gabriela Suarez, Perry R. Mitchell, Jr., Neel B. Patel, Kara Lamb, Kirpal S. Bisht*, and David J. Merkler*

Department of Chemistry, University of South Florida, Tampa, FL 33620, USA

*****Correspondence: merkler@usf.edu (D.J.M.); kbisht@usf.edu (K.S.B.); Tel.: 1-813-974-3579 (D.J.M.) or 1-813-974-0350 (K.S.B.)

Identification and Quantification of Fatty Acid Amides Produced by *Drosophila melanogaster Larvae* by LC/QTOF-MS ----- Page 2

^{1}H- and ^{13}C- NMR spectra of the fatty acid amides FA-1, FA-2, FA-3 and FA-4 -- Pages 3-10

The EIC peak and mass spectrum of the *N*-oleoylethanolamine standard -- Page 11

Targeted MS/MS spectra of OEA in *T. castaneum* extract at 20 eV collision energy displaying expected primary fragment ion, m/z 62.0602. --- Page 12

Table S1.
Identification and Quantification of Fatty Acid Amides
Produced by *Drosophila melanogaster Larvae* by LC/QTOF-MS[1]

**Fatty Acid Amide**	**Standard**		**Endogenousfrom Larvae**		**EndogenousAmount (pmoles/gram of tissue)**
	**$[M+H]^+$ *m/z***	**RT[2] (min)**	**$[M+H]^+$ *m/z***	**RT[2] (min)**	
***N*-Acylglycines**					
N-Palmitoylglycine	314.2635	5.968	314.2524	5.940	19.5 ± 7.0
N-Oleoylglycine	340.2785	6.067	340.2666	5.874	24.8 ± 12.0
N-Linoleoylglycine	338.2634	5.950	338.2399	5.794	12.1 ± 3.7
N-Archidonoylglycine	362.2630	5.800	362.2531	5.934	27.1 ± 14.7
***N*-Acylethanolamines**					
N-Oleoylethanolamine	326.2998	6.051	326.2781	6.007	1.3 ± 1.5
Anandamide	348.2840	5.768	348.2593	5.641	1.5 ± 0.8
***N*-Acyldopamines[3]**					
N-Palmitoyldopamine	392.3089	6.117	392.3356	6.237	3.8 ± 2.5
N-Oleoyldopamine	418.3243	6.196	418.3263	6.134	1.9 ± 0.5
N-Archidonoylglycine	440.3271	5.934	440.3624	5.990	2.8 ± 0.6
***N*-Acylserotonins**					
N-Palmitoylserotonin	415.3245	6.159	415.3047	6.123	2.2 ± 0.9
N-Oleoylserotonin	441.3397	6.234	441.3072	6.217	1.3 ± 0.3
N-Archidonoylserotonin	463.3319	5.976	463.3492	6.083	0.8 ± 0.4
**Primary Fatty Acid Amides**					
Palmitamide	256.2587	6.176	256.2498	6.148	20.4 ± 14.9
Palmitoleamide	254.2434	5.830	254.2322	5.805	19.2 ± 5.3
Oleamide	282.2741	6.250	282.2706	6.195	50.0 ± 31.3
Linoleamide	280.2585	5.950	280.2523	5.965	5.1 ± 4.2

[1]The fatty acid amides were extracted, analyzed, and quantified as described by Jeffries *et al*. [20].
[2]RT is retention time
[3]Identified and quantified as the *N*-acyldopamine quinone.

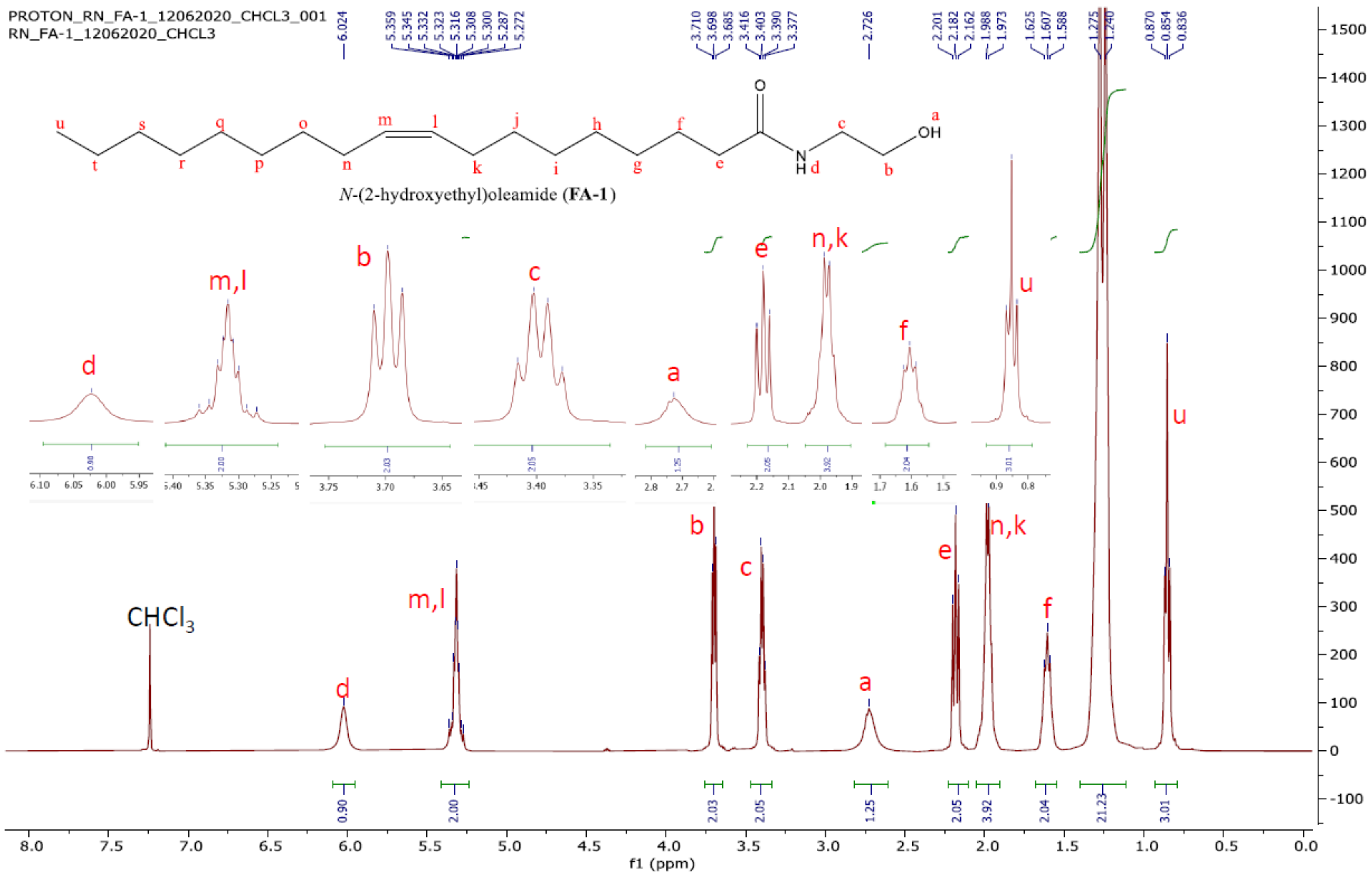

Figure S1: ^{1}H NMR (600 MHz, $CDCl_3$) of **FA-1**, the inset shows multiplicity of the signals.

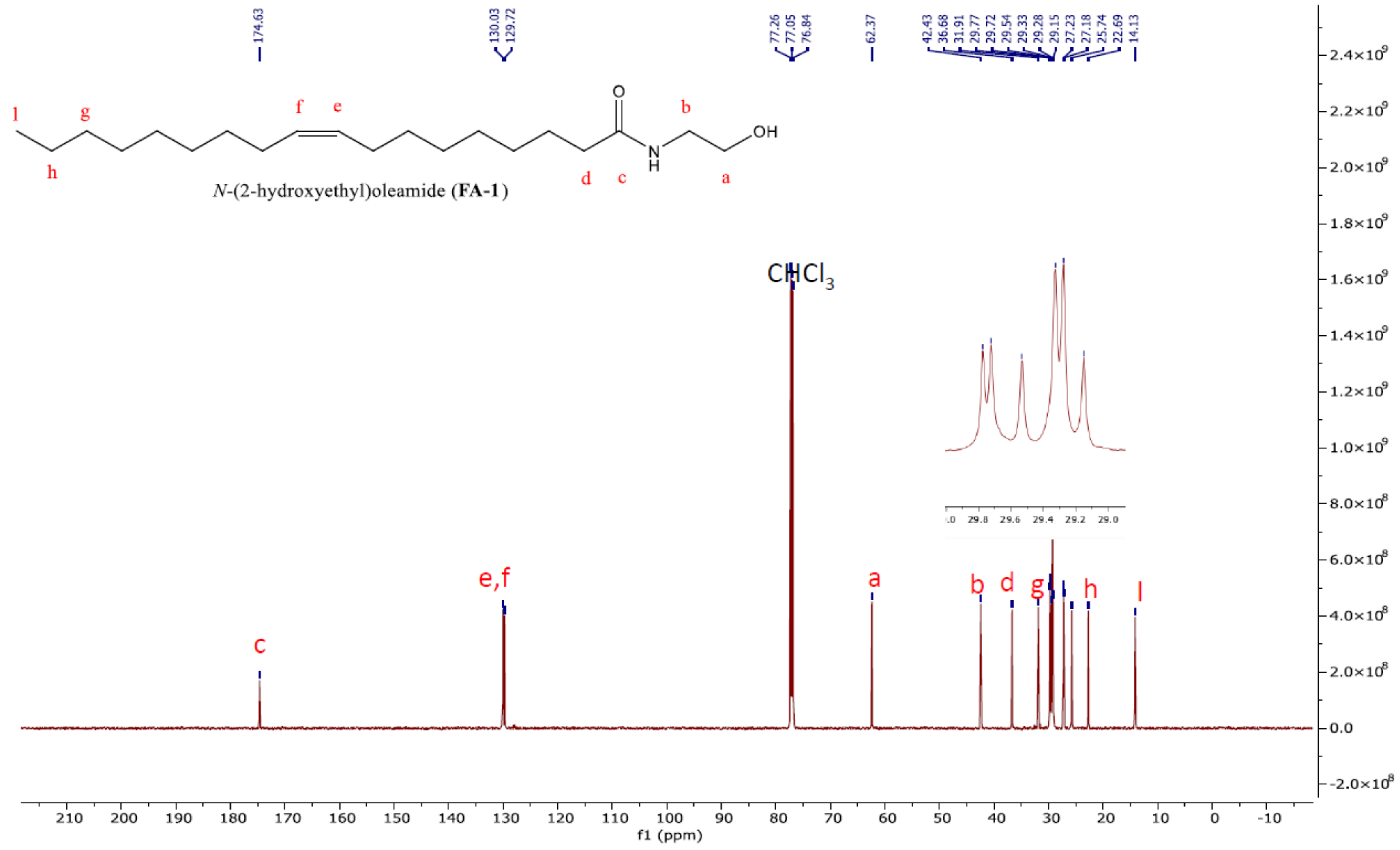

Figure S2: ^{13}C NMR (151 MHz, $CDCl_3$) of **FA-1**, the inset shows clusterd signals.

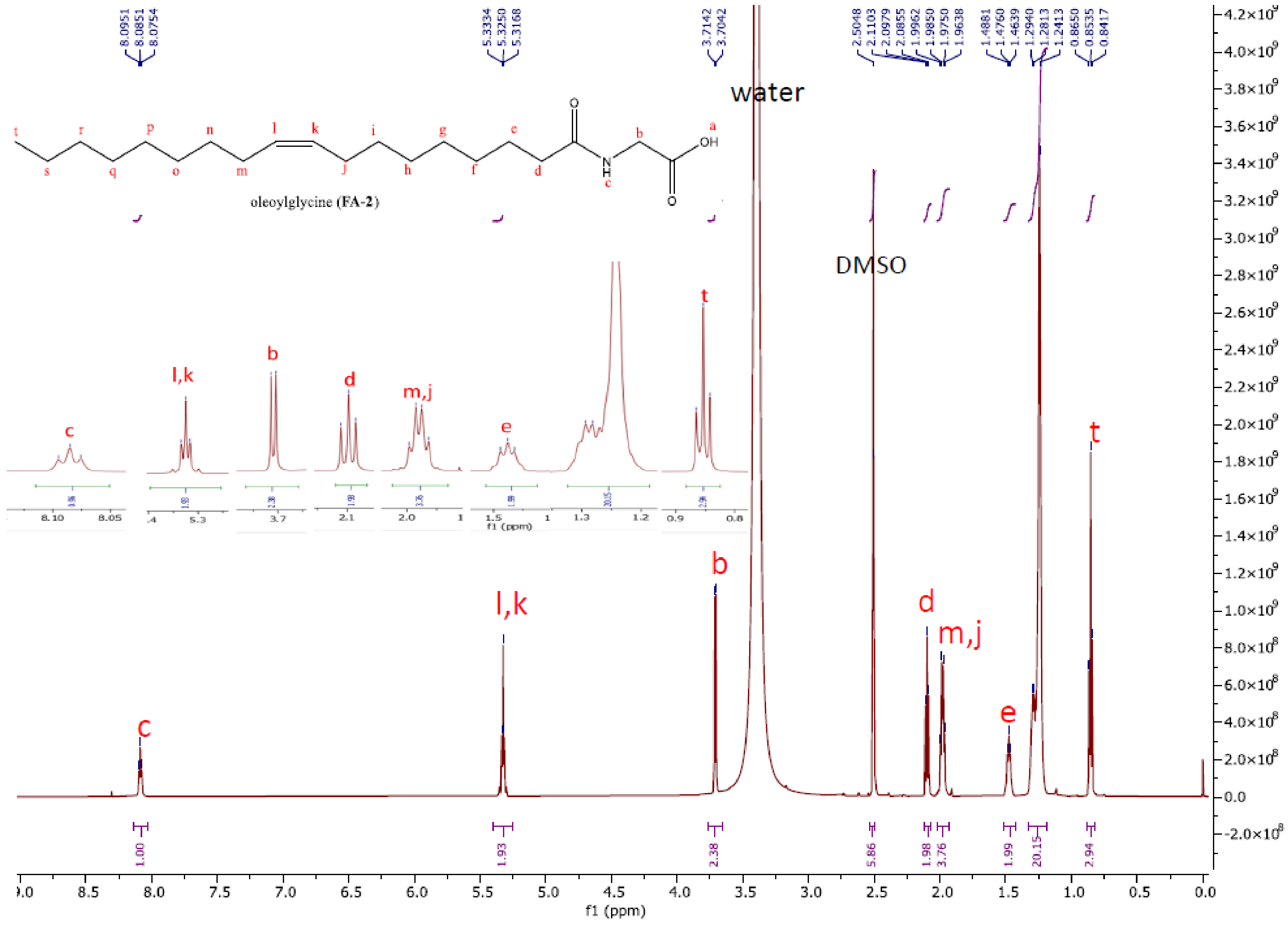

Figure S3: ^{1}H NMR (600 MHz, DMSO) of **FA-2**, the inset shows multiplicity of the signals.

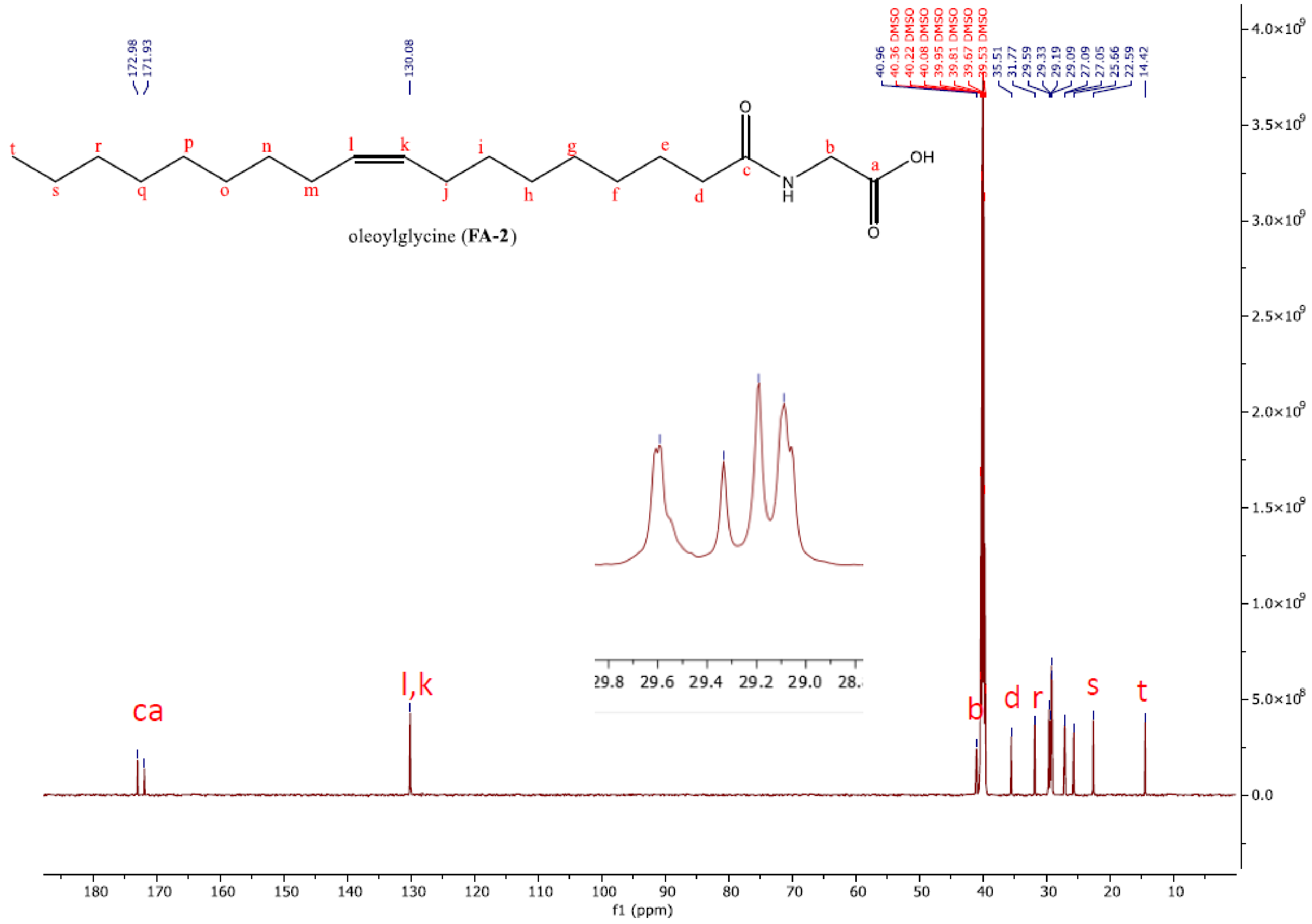

Figure S4: ^{13}C NMR (151 MHz, DMSO) of **FA-2**, the inset shows clusterd signals.

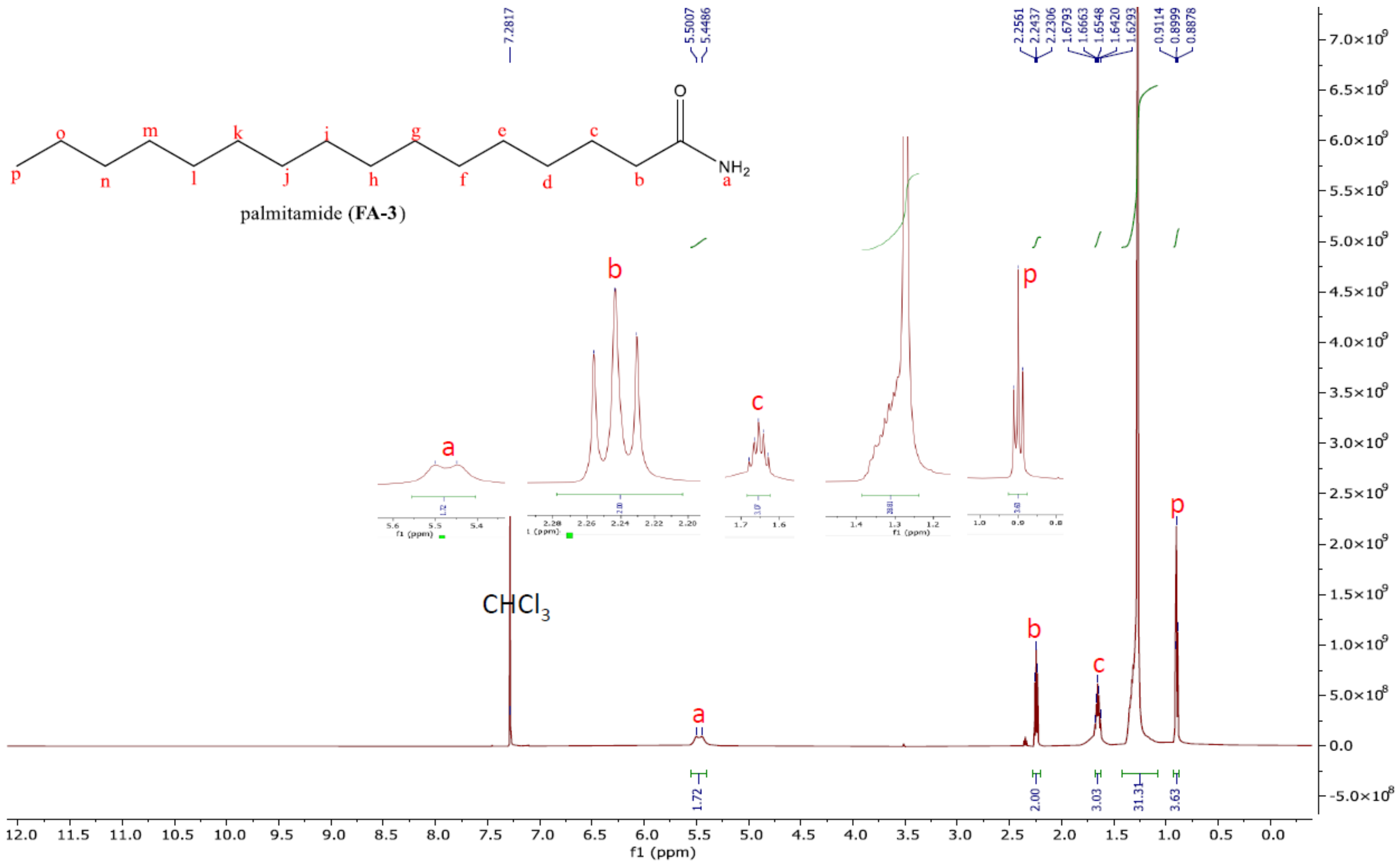

Figure S5: ^{1}H NMR (600 MHz, $CDCl_3$) of **FA-3**, the inset shows multiplicity of the signals.

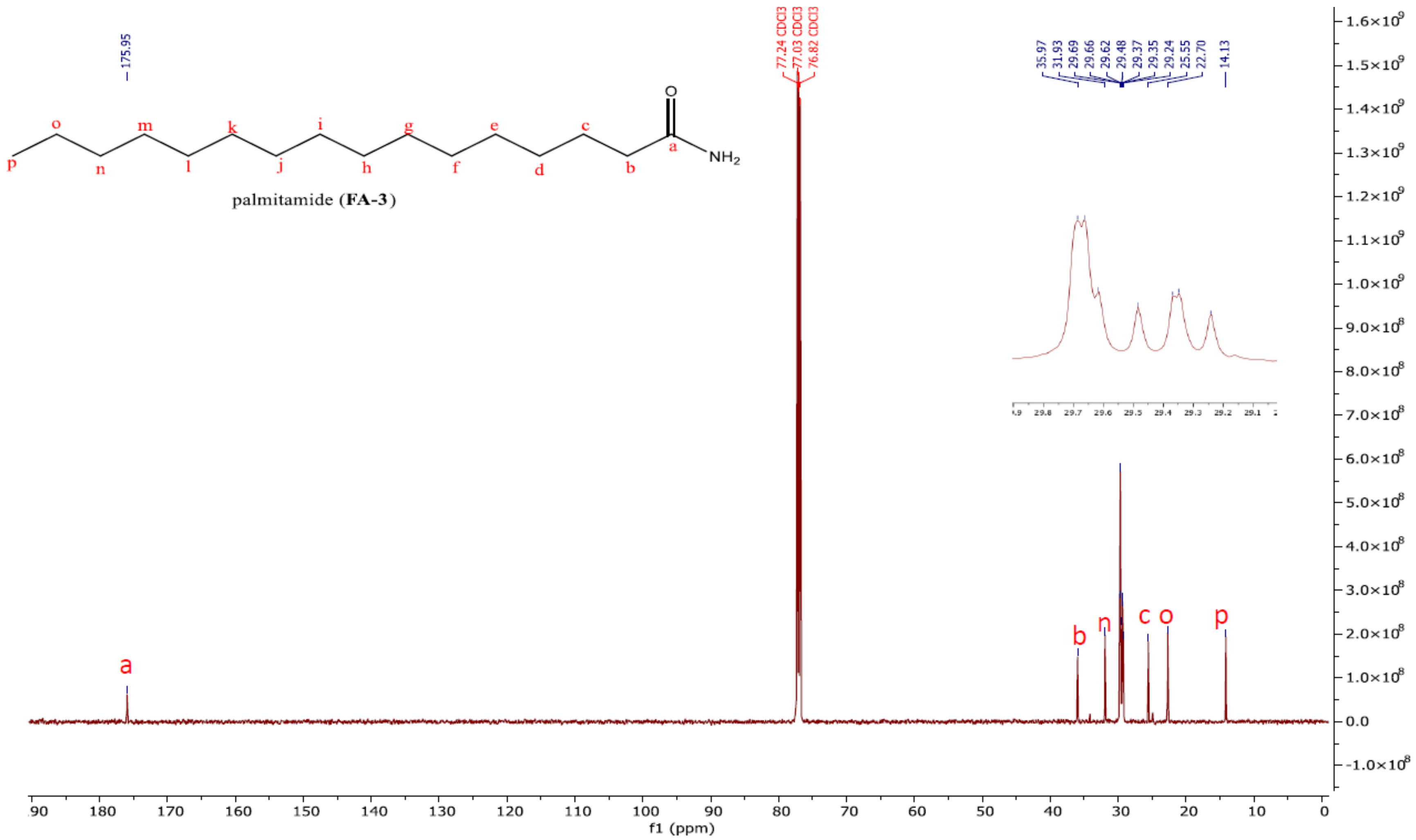

Figure S6: ^{13}C NMR (151 MHz, $CDCl_3$) of **FA-3**, the inset shows clusterd signals.

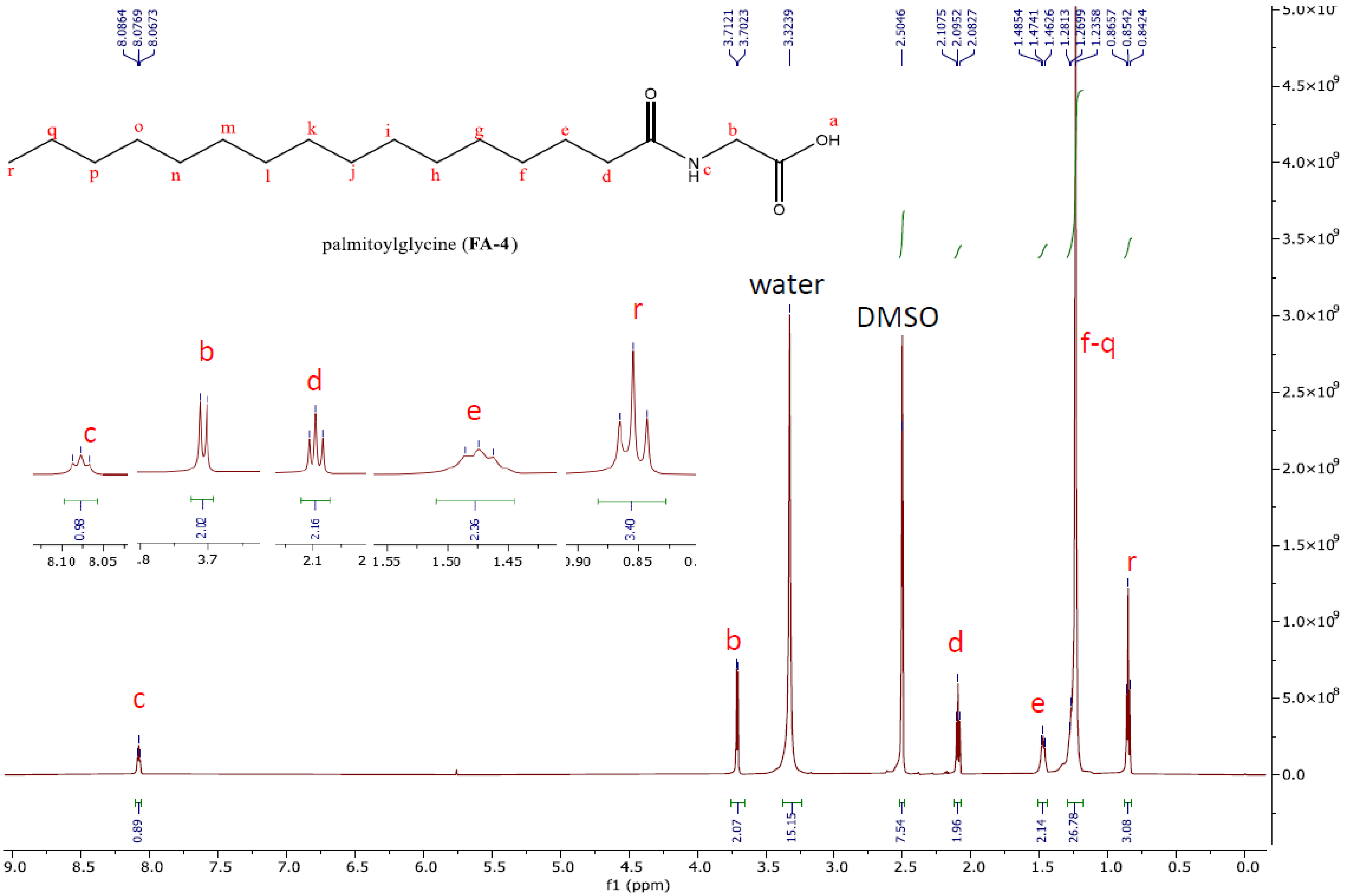

Figure S7: ^{1}H NMR (600 MHz, DMSO) of **FA-4**, the inset shows multiplicity of the signals.

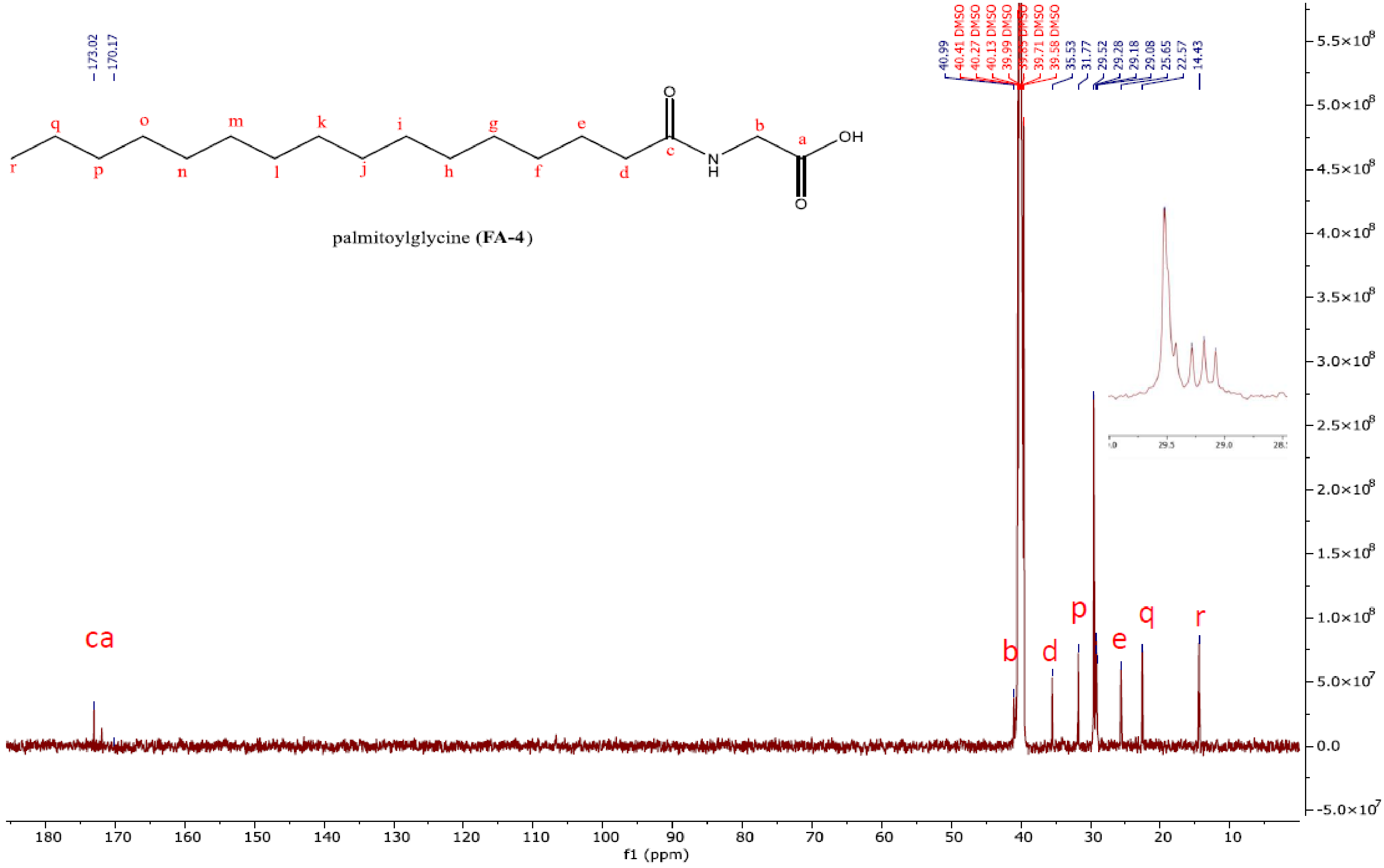

Figure S8: ^{13}C NMR (151 MHz, DMSO) of **FA-4**, the inset shows clusterd signals.

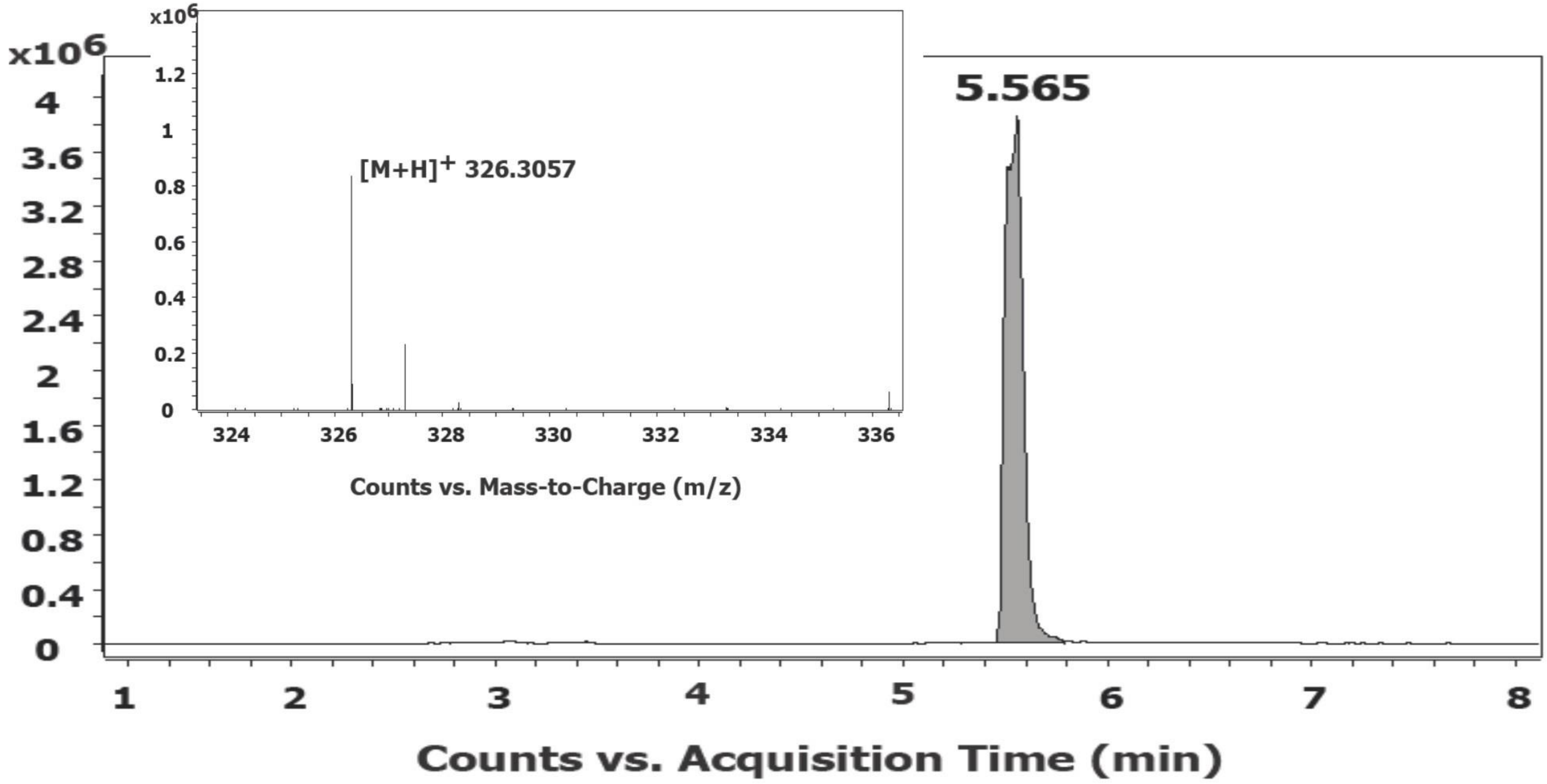

Figure S9: The EIC peak and mass spectrum of the *N*-oleoylethanolamine standard

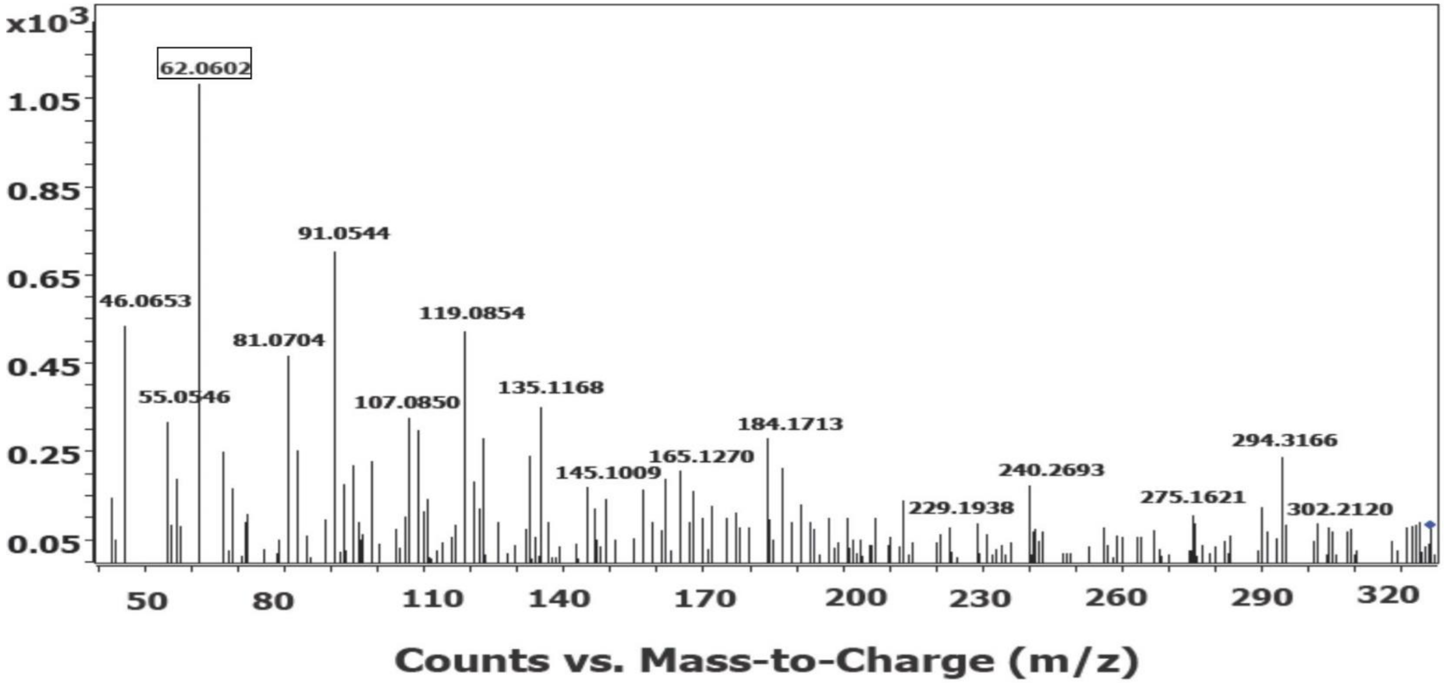

Figure S10: Targeted MS/MS spectra of endogenouse *N*-oleoylethanolamine in the *T. castaneum* extract at 20 eV collision energy displaying expected primary fragment ion, m/z 62.0602.
